# Influence of Charge Lipid Head Group Structures on Electric Double Layer Properties

**DOI:** 10.1021/acs.jctc.1c00800

**Published:** 2021-12-22

**Authors:** Klemen Bohinc, Mario Špadina, Jurij Reščič, Naofumi Shimokawa, Simone Spada

**Affiliations:** † Faculty of Health Sciences, University of Ljubljana, SI-1000 Ljubljana, Slovenia; ‡ Faculty of Chemistry and Chemical Technology, University of Ljubljana, Večna pot 113, SI-1000 Ljubljana, Slovenia; ¶ Japan Advanced Institute of Science and Technology, 1-1 Asahidai, Nomi, Ishikawa 923-1292, Japan; § National Institute of Oceanography and Applied Geophysics - OGS, 34010 Trieste, Italy

## Abstract

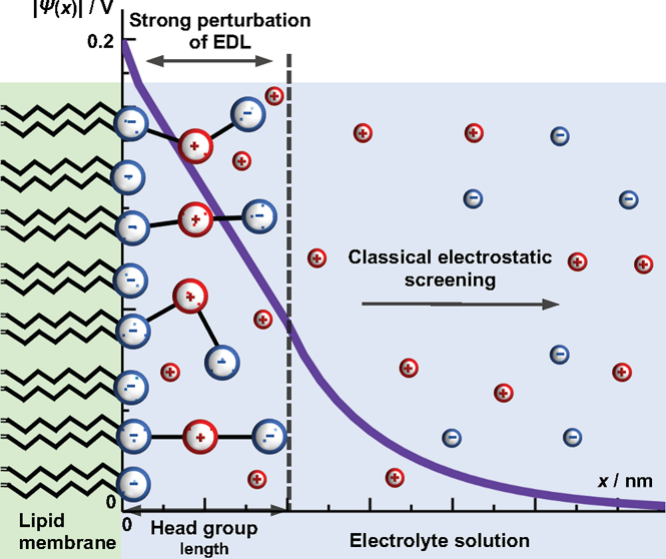

In this study we derived a model for a multicomponent lipid monolayer in contact with an aqueous solution by means of a generalized classical density functional theory and Monte Carlo simulations. Some of the important biological lipid systems were studied as monolayers composed of head groups with different shapes and charge distributions. Starting from the free energy of the system, which includes the electrostatic interactions, additional internal degrees of freedom are included as positional and orientational entropic contributions to the free energy functional. The calculus of variation was used to derive Euler–Lagrange equations, which were solved numerically by the finite element method. The theory and Monte Carlo simulations predict that there are mainly two distinct regions of the electric double layer: (1) the interfacial region, with thickness less than or equal to the length of the fully stretched conformation of the lipid head group, and (2) the outside region, which follows the usual screening of the interface. In the interfacial region, the electric double layer is strongly perturbed, and electrostatic profiles and ion distributions have functionality distinct to classical mean-field theories. Based purely on Coulomb interactions, the theory suggests that the dominant effect on the lipid head group conformation is from the charge density of the interface and the structured lipid mole fraction in the monolayer, rather than the salt concentration in the system.

## 1 Introduction

In biological systems, electrostatic interactions between charged lipid membranes and aqueous electrolyte solutions are of fundamental interest.^1^ The membrane has a bilayer structure mainly consisting of lipid molecules. The lipids are amphiphilic molecules, which consist of two dissimilar parts.^2^ One part is hydrophobic and is referred to as the tail. The second hydrophilic part is directed toward the aqueous solution in contact.^3^

Some physicochemical phenomena in lipid membranes have been investigated in association with biocellular functions. In multicomponent lipid membranes, the compositional heterogeneous structure known as phase separation emerged spontaneously at lower temperature.^4−6^ The formation mechanism and stability of phase separation in lipid membranes have been discussed in relation to the raft structure, which is believed to be formed in plasma membranes and to play important roles in signal transduction and membrane trafficking.^7,8^ In the past decade, the phase separation in negatively charged lipid membranes has been attracting experimental attention.^9−12^

Lipid head groups can either carry an excess charge or be zwitterionic.^3^ The charge of the head groups, and thus the charge of the bilayers, depends on the pH, the type of salt, and its concentration.^13^ The most common naturally occurring anionic lipids are phosphatidylserine (PS) and phosphatidylglycerol (PG).^14^

Around pH = 7, both anionic lipids have charges, with charge number −1. PS has a negatively charged phosphate group, a positively charged amine group, and a negatively charged carboxy group from the part closest to the hydrophobic group (see the structure of the PS ion in Figure 2, below). In contrast, PG has a negatively charged phosphate group, and the terminal group (glycerol) does not readily dissociate (electroneutral).

Cationic lipids are used for artificial membranes.^15^ The head groups of uncharged lipid molecules are zwitterionic, typically with two opposite elementary charges separated by a few angstroms.^3^ Common zwitterionic lipids are phosphatidylcholine (PC), phosphatidylethanolamine (PE), and sphingomyelin (SM). For example, the head group of the lipid PC is composed of a negatively charged phosphate group separated from the choline unit that carries a positively charged nitrogen.^14^ The head group of the lipid PE is composed of a negatively charged phosphate group separated from the ethanolamine group that carries a positively charged nitrogen.

Apart from synthetic membranes, multivalent, negatively charged lipids are present in biological membranes. Phosphatidylinositol (PI) is a monovalent, negatively charged lipid, with the negative charge being the phosphate group. For example, PI works as a substrate for the production of the second messenger in intracellular signaling pathways.^16,17^ Diacylglycerol (DAG) and inositol triphosphate (IP_3_) are produced by phospholipase C from PIP_2_, in which two positions of the inositol ring of PI are phosphorylated. In addition, it is involved in intracellular transport,^18^ membrane deformation,^19,20^ regulation of organization, and dynamics of actin cytoskeleton,^21^ and control of intracellular signaling pathways.^22^ In the expression of these functions, the phosphorylation process of the inositol ring of PI is important, and the phosphorylated PI becomes a multivalent negatively charged lipid; PIP is phosphorylated at one position, PIP_2_ at two positions, and PIP_3_ at three positions. The latter ones are especially interesting from an electrostatic point of view, due to multiple charges per surface area of the molecule in the bilayer.

While the structural aspects of lipid membranes were theoretically extensively studied,^23−26^ the description of the larger system, such as the charged membrane in contact with an electrolyte solution, is often simplified in order to reduce computational resources. The ions of the electrolyte redistribute around the charged bilayer (or the monolayer to simplify) and form an electric double layer (EDL).^27,28^ The way to assess the properties of the EDL is to use mean-field theories.

The majority of mesoscopic modeling approaches for lipid monolayers, and colloidal systems in general, assume that the interface between hydrophilic head groups and the aqueous solution of ions is a flat surface with a given surface charge density.^29−31^ In most cases the “bare” charge of lipid monolayers is high, and classical continuum mean-field Poisson–Boltzmann (PB) theory is close to the limit of providing an adequate description of electrostatic properties of the system.^32,33^ Despite the issue, it was shown by molecular dynamics (MD) simulations that, for the majority of charged lipid assemblies, ions can penetrate inside the “soft” interfacial region composed mostly of head groups.^34−36^ Counterions embedded into head groups partially neutralize the “bare” charge of lipid assemblies. There are several ways of accounting for ion partitioning and resulting screening of bare charge. First, one can consider ion partitioning in the interfacial region as a constant.^29,34,37^ It can be estimated either from experiments (more qualitative value) or as a fitted parameter. The second approach is to use the most common colloidal approximation—the Stern layer.^35^ While this assumes the lipid monolayer is a rigid, impermeable wall, again the counterions’ adsorption coefficient lowers the bare charge, and the resulting apparent surface charge density is much lower. The extension of the Stern layer concept is the multicomponent charge regulation method that considers particular charged segments of lipid head groups explicitly.^30^ It must be emphasized that almost all of these approaches consider that ion adsorption occurs within a certain thickness, which is sometimes even called the “active diffuse interface”.^38^ The thickness of this interfacial region is approximately equal to the length of maximum extension of the head group along the vector normal to the interface. The result of the aforementioned approaches is that the overall lipid membrane has lower surface charge density. In those conditions, the electrostatic potential in the aqueous solution can be described even by a simple Gouy–Chapman model. Still, there are always two or more fitted parameters involved in models.

Classical density functional theory (cDFT) offers a general framework which can adopt many modifications to provide more realistic descriptions of lipid membrane systems. Already existing modifications of PB theory for electrolytes in nanoconfinement include the structure and the spatial distribution of charge for both ions and solvent.^39−45^ The local dielectric properties of the aqueous phase can be explicitly included by considering Langevin dipoles.^46^ Apart from the upgrades of the solution, the structure of the interface was, up to now, accounted for in only a few attempts.^47,48^ When upgrading the interface with the structure of lipid head groups, the first modification is to introduce the terminal charge of the lipid that is now immersed into the liquid and has an angular degree of freedom, as was proposed in the case of zwitterionic lipids.^47−49^ Further upgrades can have additional coarse-grained elements of lipid structure in a monolayer, such as the flexibility or even different charges and shapes of head groups. The system of negatively charged lipids, such as in the case of PS or PIP_2_, has additional rotational degrees of freedom of charged segments, beyond that of the simpler zwitterionic case. To the best of our knowledge, negatively charged lipid monolayers with flexibility of head groups were not modeled on purely theoretical grounds, but only molecular simulations were used to estimate the charge density profiles.^50^ Furthermore, in the case of mixed lipid monolayers, the mole fraction of head groups with additional degrees of freedom is an important contributor to the electrostatic properties. Indeed, better prediction of EDL properties is pivotal for understanding many phenomena, such as ion partitioning and the thickness of the interfacial region,^38^ or the lateral interactions between head groups that govern in-plane phase separations.^51^

Apart from cDFT, Monte Carlo (MC) simulations were often used to test theoretical predictions.^23,52^ By MC simulations, various static properties can be deduced, including the thermodynamics and structure of model systems.^49^ While molecular dynamics (MD) simulations can be used to include further effects into the description of the interface wetted with water molecules and the soft-matter aspect of the interface,^50,53,54^ the computational demand is very high.^36^ In fact, both MC and MD simulations are limited to a small range of system compositions, e.g., high salt concentrations, small patches of predetermined mole fractions of lipids in the bilayers, etc. This generates a demand for mean-field and general cDFT theories which can be utilized to recover thermodynamics of lipid systems for various system compositions and applications.^43^

In this article we will demonstrate a model for a multicomponent monolayer of surface-bound lipid head groups immersed in an aqueous solution of ions. Lipid head groups have a particular structure and discrete charge distribution. The free energy functional includes the electrostatic interactions and additional internal degrees of freedom included as positional and orientational entropic contributions. The model is developed for rod-like, flexible, and triangular lipid head group structures. The free energy of the system is derived, and the minimization procedure is applied in order to determine the equilibrium charge distributions and the preferential orientation of lipid head groups with respect to the interface. The theoretical results are verified with MC simulations.

## 2 Model and Methods

**Figure 1:**
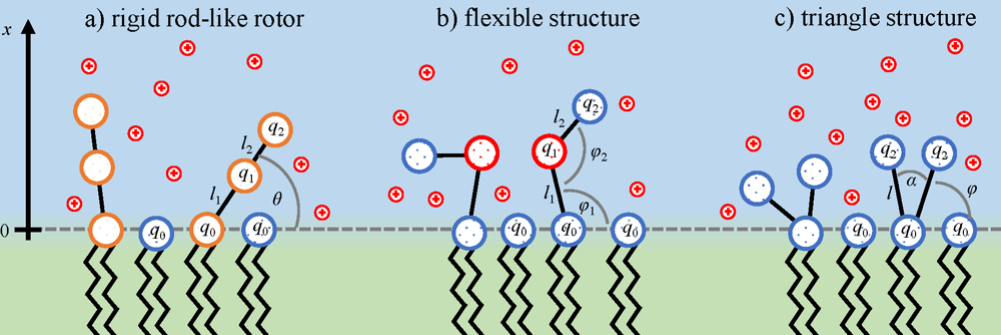
Schematic representation of the lipid monolayer in contact with the aqueous solution. Mobile ions are point-like charges and are represented by small red circles in the sketch. The monolayer is composed of two types of lipids. The first components are structured lipids with spatial charge distribution. We consider three cases: (a) rigid rod-like, (b) flexible segments, and (c) triangular head groups. The other component of the monolayer is a lipid that has its head group treated in a simplified manner, as only a point-like charge, and is expressed through the mole fraction of lipids in monolayer as β. It contributes to the overall charge density of the interface. In all cases of structured lipids, the first segment is immobile, lies in the plane *x* = 0, and has the charge number *q*_0_. Other segments protrude into the aqueous solution and possess certain degrees of freedom (see Model and Methods). Structures represent actual lipids, see Figure 2.

The generic structure for the lipid bilayer (the membrane) is a body of low dielectric constant, whereas the electrolyte solution represents a body of high dielectric constant. At the interface of both bodies, the charges of the membrane are embedded in the electrolyte solution, see Figure 1. The charges can be fixed or mobile around some fixed position at the interface.^27^ We consider only one lipid monolayer. A Cartesian coordinate system is chosen whose *x*-axis is oriented perpendicular to the charged surface located at *x* = 0. Due to sufficiently large planar surfaces and the translational invariance of the system along the *y* and *z* directions, we can describe the system with functions depending only on the *x*-coordinate.

**Figure 2:**
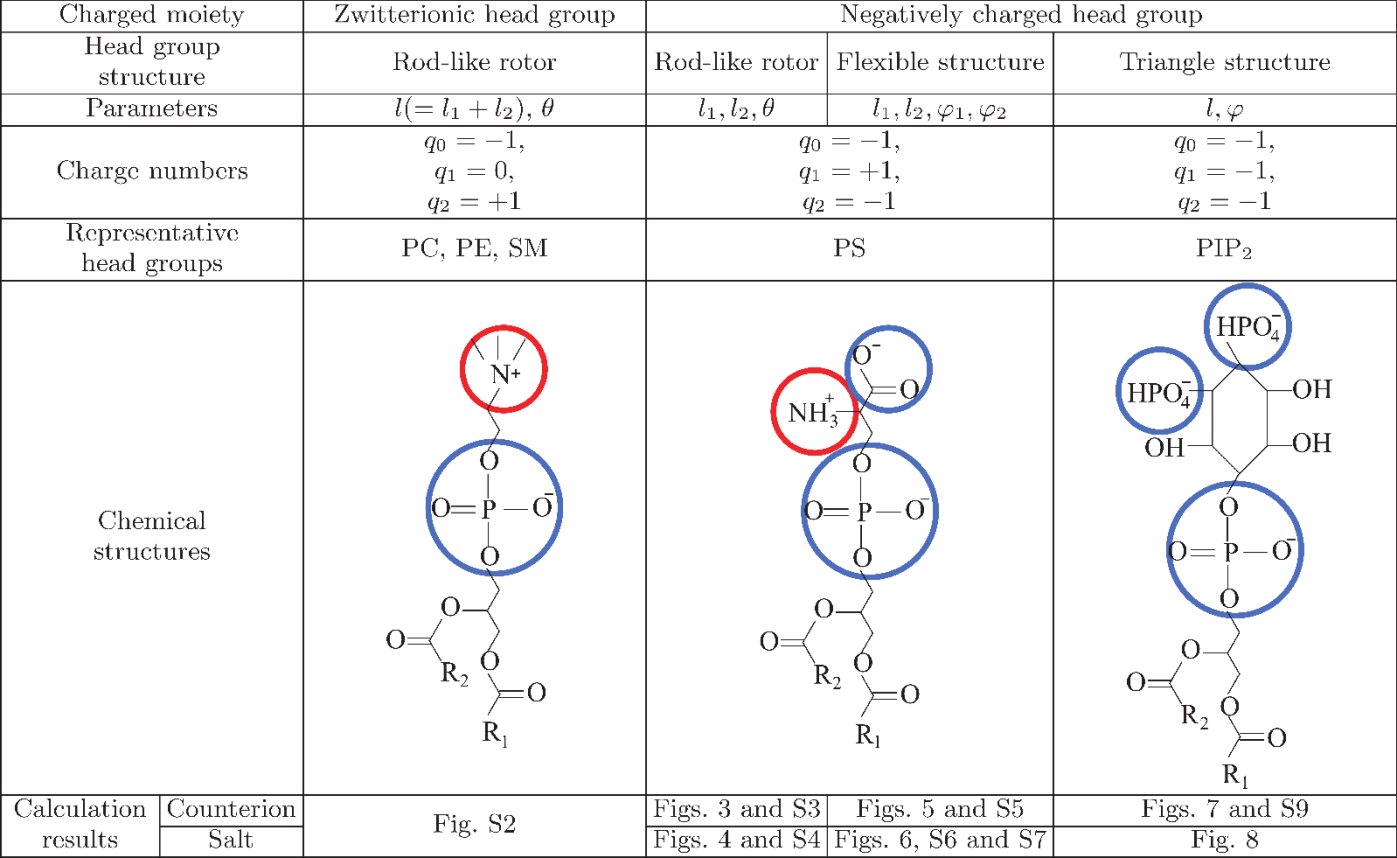
Schematic representation of different types of head groups: zwitterionic (second column) and negatively charged head groups, where we further distinguish rigid rotors and flexible head groups (third column), and triangular head groups (the fourth column). *q*_0_, *q*_1_, and *q*_2_ are the charge numbers of the particular structure. For all cases, *q*_0_ represents the phosphate group, depicted by a large blue circle. The chemical structures of typical head groups are presented. In this work, we present the theory for the modeling of negatively charged lipids: rigid rotors, flexible head groups, and triangular head groups. The theory and MC simulations of zwitterionic rod-like head groups are presented in the Supporting Information only as a validation of the approach since the theoretical descriptions for these systems are already known from the literature.^47,48^

The surface area per lipid molecule is *a*. For the sake of simplicity, we did not include charge regulation of lipids by pH of the solution. As a result, we consider all lipid segments charged. Nevertheless, the charge regulation can be included via association constants for each charged group. In the case of lipids considered in this work, head groups can have up to three charges. The initial charge with the charge number *q*_0_ lies in the plane *x* = 0, and it represents the phosphate group in all cases. The lipids with only one charge, without the structure modifications (such as PG and PI), are represented through the parameter β, which is defined as the mole fraction of structured charged lipids in a multicomponent monolayer. PS, PE, PC, and SM are represented by two distinct models: the rigid rotor and flexible structures (see third column in Figure 2). PIP_2_ is represented by the model of a triangular head group (see fourth column in Figure 2). The surface charge density is σ = *q*_0_*e*/*a*, where *e* is the elementary charge.

As shown in Figure 1, we consider three cases. In the first case (a) the head group has a linear rigid structure. The second case (b) corresponds to the flexible head group, whereas in the third case (c) the head group has a triangular structure. The bond lengths between the segments are assumed to be fixed. Two rigid rod segments are linked together. The first segment has a length *l*_1_, whereas the second segment has a length *l*_2_. The rod-like segments are orientationally mobile. The second charge, located at the flexible joint between two rod-like segments, has charge number *q*_1_. The third charge, located at the mobile terminus, has charge *q*_2_. The bond lengths are taken to represent all conformations excluding vibrations.

The properties of these three distinct cases of lipid head groups are summarized in Figure 2. Furthermore, generic structures are assigned to actual lipids.

### 2.1 Free Energy Functional

The total free energy *F* of the system can be written as
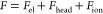
1The electrostatic energy *F*_el_ is equal to
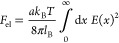
2where *E* is the reduced electric field strength. The Bjerrum length, defined as *l*_B_ = *e*^2^/(4πϵ_w_ϵ_0_), denotes the distance between two elementary charges at which their Coulomb interaction equals the thermal energy *k*_B_*T*, where ϵ_w_ is the dielectric constant of the aqueous solution and ϵ_0_ is the permittivity in free space. *k*_B_ is Boltzmann’s constant, and *T*is the absolute temperature. *T* is set to 298 K, and ϵ_w_ = 80, which yields the Bjerrum length *l*_B_ = 0.7 nm. The integration over *x* runs in the half space between 0 and *∞*.

The entropic contribution of head groups to the free energy *F*_head_ is
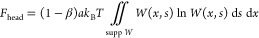
3where *W*(*x*, *s*) is the probability density function, *x* represents the location of the charge *q*_1_ projected to the *x*-axis, and *s* represents the location of the terminal charge *q*_2_ on the *x*-axis. The fraction of non-structured lipids is given by β. The region of integration is given by
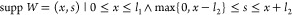
4
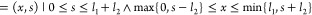
5*F*_head_ is the additional term in the free energy functional, compared to the one that considers only *F*_ion_ terms and yields the PB level of description. *F*_head_ represents the upgrade of the interfacial region within the cDFT framework.

The expression of the ideal demixing free energy, *F*_ion_^salt^, of a two-component ideal gas, composed of mobile counterions and co-ions, is
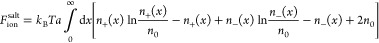
6where *n*_+_ and *n*_–_ are concentrations of counterions and co-ions, respectively. Ions are point-like charges with no volume correlations. Far away from the lipid layer, both concentrations *n*_+_ and *n*_–_ adopt identical bulk values *n*_0_. Hence, in a bulk system, the free energy is zero.

For the system containing only counterions, the ideal mixing free energy, *F*_ion_^ct^, is
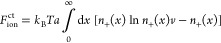
7where *n*_+_ is the concentration of counterions and *v* is the de Broglie parameter.

There are two constraints. The first is Gauss’s law, and the second is the normalization condition of the probability density function:
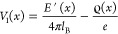
8
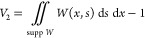
9where ϱ is the volume charge density, given by the mobile ions and mobile part of the head groups. The prime ′ denotes the first derivative with respect to *x*.

The boundary conditions are given at the charged surface and in the bulk:
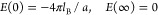
10

### 2.2 Euler–Lagrange Equations

The functional of the system *I* can be written as
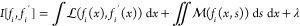
11where λ is a constant and *f*_*i*_ are the functions: the electric field strength *f*_1_ = *E*, the probability density function *f*_2_ = *W*, and the concentrations *f*_3,4_ = *n*_+,–_. In the case of counterions only, *f*_3_ = *n*_+_ and *f*_4_ = 0. The prime ′ denotes the derivation with respect to *x*. The corresponding Euler–Lagrange equations are
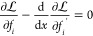
12
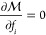
13

The Lagrange density of the system of lipid head groups and counterions is given by
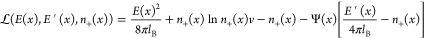
14
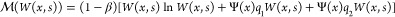
15where Ψ is the Lagrange parameter.

### 2.3 Rigid Lipid Head Groups

In rigid lipid head groups, the two segments of length *l*_1_ and *l*_2_ are rigidly connected at an angle of 180°. The head group can freely rotate only about the surface-bound charge group *q*_0_ located at the plane *x* = 0. In addition, we assume that only counterions are present in the solution. The probability density function depends only on the coordinate *x*. The Euler–Lagrange equations deliver
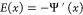
16
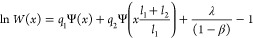
17
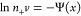
18The constraint is given that *E*′(*x*) = 4*πl*_B_ϱ(*x*)/*e*, where the volume charge density is
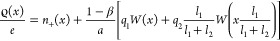
19

### 2.4 Flexible Lipid Head Groups

For the system of flexible lipid head groups and only counterions, the Euler–Lagrange equations deliver
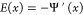
20
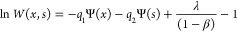
21
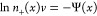
22The constraint is given that *E*′(*x*) = 4π*l*_B_ϱ(*x*)/*e*, where the volume charge density is
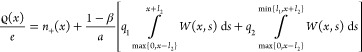
23

### 2.5 Triangular Lipid Head Groups

In lipid triangular head groups, one charge is attached in the plane *x* = 0. Two charges, *q*_1_ and *q*_2_, lie in the solution. The distance between the charge in the plane *x* = 0 and one of the two charges in the solution is equal to *l*. The angle between the lines connecting the charges in the solution with the charge at *x* = 0 is equal to α. We assume that charges can move only within one plane. The Euler–Lagrange equations deliver
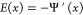
24
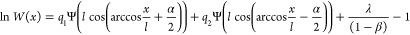
25
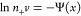
26The constraint is given that *E*′(*x*) = 4π*l*_B_ϱ(*x*)/*e*, where the volume charge density is
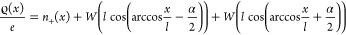
27

### 2.6 Numerical Implementation

The Euler−Lagrange equations have been solved with a finite dierence method.

In the case of rigid and flexible lipid head groups (sections 2.3 and 2.4), the domain [0,+∞) has been divided into three sub-regions, namely *R*_1_ = [0, *d*_2_], *R*_2_ = [*d*_2_, *d*_1_], and *R*_3_ = [*d*_1_, +∞), with *d*_1_ = *l*_1_ + *l*_2_ and *d*_2_ = *l*_1_. The first two regions have been discretized with a fixed step *h*, and the solution has been computed numerically, whilr in the third one the solution has been calculated analytically after replacing the electro-neutrality boundary condition (i.e., Ψ′(+∞) = 0) with the analytical constraints,
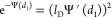
28or
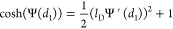
29for the counterions case and the salt case, respectively.

In order to obtain meaningful electric field values, the potential Ψ has been chosen to be in the class C^1^ ([0, +∞])) of the continuous functions with continuous first derivative. Thus, the continuity of the first derivative when passing from region *R*_1_ to *R*_2_ requires the condition
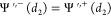
30where Ψ′^,–^ and Ψ′^,+^ represent the left and right derivatives of Ψ. Note that condition 28 or 29 is already sufficient to preserve the continuity of the first derivative in *d*_1_, i.e., in the intersection between *R*_2_ and *R*_3_.

The case of triangular lipid heads groups (section 2.5) is similar to the previous cases, but the boundaries of the three sub-regions are defined by *d*_1_ = max{*l*_1_, *l*_2_} and *d*_2_ = min{*l*_1_, *l*_2_}. Note that if *l*_1_ = *l*_2_, then region *R*_2_ collapses and the problem simplifies, making constraint 30 no more necessary.

After the discretization, the value of Ψ between grid points is approximated by linear interpolation, while the first and second derivatives of Ψ at the grid points have been approximated by a second-order finite difference operator, i.e.,
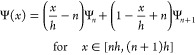
31
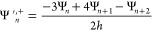
32
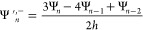
33
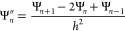
34where Ψ_*n*_ = Ψ(*nh*) is the discretization of Ψ.

The integrals in eqs 9 and 23 have been estimated by the trapezoidal rule. The resulting nonlinear system has been solved with MATLAB using the *fsolve* function.

### 2.7 Monte Carlo Simulations

The structures studied by MC simulations are the same as theoretical ones (zwitterionic, rigid rotor, flexible, and triangle structures). As in the theory, the negative charges of head group segments at *x* = 0 are treated as having uniform surface charge density, σ. The difference in the MC simulations is the fact that all charged species are considered within the primitive restrictive model (i.e., rigid spheres with charge located in the center).

The model was treated by canonical MC simulations using the integrated MC/MD/Brownian dynamic simulation package Molsim,^52^ following the standard Metropolis scheme.

The simulation box, which mimics a charged slit, was a rectangular parallelepiped with periodic boundary conditions applied in (*x*, *y*) directions. In the case where the aqueous solution has only counterions as mobile ions, their total charge as well as the positive parts of zwitterions were compensated by the negative surface charge, rendering the system electroneutral.

Each lipid layer was represented by 400 lipid molecules, including fully ionized ones. A typical MC box size was 15.491 9 × 15.491 9 × 8 nm at the highest surface charge density (*a* = 0.6 *e*/nm^2^) and 48.989 8 × 48.989 8 × 8 nm at lower surface charge density studies (*a* = 6 *e*/nm^2^).

Interparticle interactions were calculated as in ref (55). For mobile counterions, each trial move consists of random displacement in all three directions. Zwitterions and negatively charged head groups can perform translations on the surface and random rotations of charged segments.

Displacement parameters were chosen to ca. 50% of the acceptance rate. A total of 20 000 attempted moves per particle were used for the equilibration phase followed by 100 000 attempted moves during production runs. Distribution of particles along *x*-axis was collected using a bin width of 0.02–0.1 nm.

## 3 Results and Discussion

The primary focus of the present work is to investigate the structure of the EDL around the phospholipid monolayer. The cases that we will present include the monolayer in contact either with counterions only or with the salt of monovalent ions. As in the Model and Methods section, we will consider the rigid rod-like, flexible rod-like, and triangular shapes of head groups. Again, it is worth emphasizing that the structure of head groups is considered as an additional entropic contribution to the free energy, *F*_head_, with specifically defined probability density function *W* for each of the three head groups. The detailed charge distribution of lipid groups is taken into account in the volume charge density which appears in Gauss’s law. Monovalent ions are included via ideal free mixing entropy. The minimization of the free energy is performed by Euler–Lagrange equations which are solved numerically. In the following three subsections, we present and discuss the numerical results. The minimal model parameters were chosen to approximate lipid molecules described in the previous section (see Figure 2). To simplify the discussion and to demonstrate the technical part of this cDFT approach (to date, the new approach), we did not perform a rigorous parametrization of the model. This will be the subject of future publications, where we will focus on thermodynamic aspects of particular lipids. For all presented calculations, the Bjerrum length considered is equal to *l*_B_ = 0.7 nm, and the mole ratio of non-structured lipid head groups is equal to β = 0.5. Note that we made additional calculations for different β values to demonstrate the generality of the method, and the results are given Figure S3 in the Supporting Information.

### 3.1 Rigid Lipid Head Groups

For the case of rigid rotors, the first negative charge is fixed at *x* = 0 with the charge number *q*_0_ = −1. The middle charge *q*_1_ = +1 within the rod is located at the distance *l*_1_ from the fixed charge, whereas the terminal charge *q*_2_ = −1 is located at the distance *l*_1_ + *l*_2_ from the fixed charge. The corresponding probability density function *W*(*x*) for the middle charge *q*_1_ refers to the projection *x* on the *x*-axis (perpendicular to the interface; see Figure S1 in the Supporting Information).

**Figure 3:**
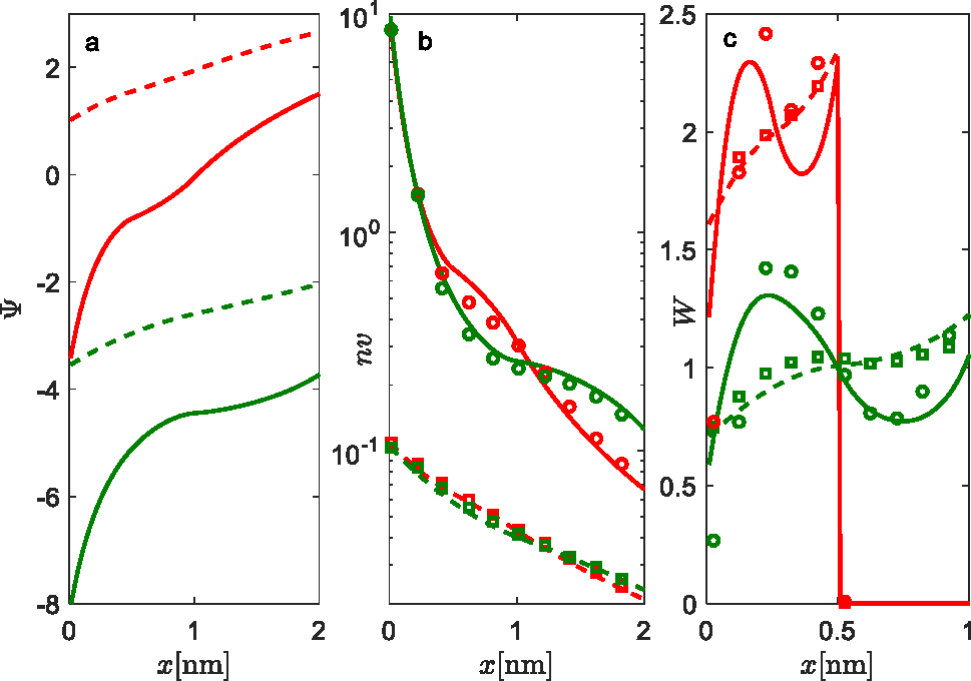
Properties of EDL with rigid rod-like lipid head groups in contact with counterions only. (a) Electrostatic potential profiles Ψ, (b) concentration profiles of counterions *n*, and (c) probability densities of the middle charge *q*_1_ for the rigid rod-like head groups *W*. The lengths of the head groups are *l*_1_ = *l*_2_ = 0.5 nm (red lines) and *l*_1_ = *l*_2_ = 1 nm (green lines). Results are given for two surface areas per lipid molecule (the two charge densities of the interface): *a* = 0.6 nm^2^ (full lines, high charge regime) and *a* = 6 nm^2^ (dashed lines, low charge regime). Only counterions are in the solution, and β = 0.5. MC simulations: *a* = 6 nm^2^ (open squares) and *a* = 0.6 nm^2^ (open circles). The system is electroneutral.

First the effect of the lengths *l*_1_ and *l*_2_ at a high surface charge density, *a* = 0.6 nm^2^, which corresponds to σ ≈ 0.3 C m^–2^ (full lines) is addressed. Note that surface area per lipid molecule *a* = 0.6 nm^2^ is somewhat an average value at ambient room temperature for many types of lipids.^54,56^Figure 3a shows the electrostatic potentials profiles Ψ for the rigid head groups which consist of three charges in contact with the solution of positive counterions only. The potential profile shows non-standard behavior of Ψ within the head group region (up to *l*_1_ + *l*_2_), whereas outside the head group region it has analytical form and follows standard PB theory. As expected, in the case of shorter head group lengths *l*_1_ = *l*_2_ = 0.5 nm (red line), the screening of the interface is achieved at shorter distances. Concentration profiles of counterions are monotonously decreasing for both *l*_1_ = *l*_2_ = 0.5 nm and *l*_1_ = *l*_2_ = 1 nm with increasing *x*, as shown in Figure 3b. At the position of the terminating negative charge segment, for both cases (segment lengths), there is a less steep decrease of counterion concentrations that looks like a “bump” in ion profiles. The conditional probability *W* for the orientation of head groups in the strongly charged phosphate layer shows a non-monotonic distribution (Figure 3c). The tendency of head groups is to approach the interface (the first maximum) due to the fact that the middle charge is contributing to screening, while the terminating charge is repelled from the interface by Coulombic repulsion. The reason for the oscillating profile is a very strong screening of the strongly charged surface by counterions. This “bump” in counterion density profiles is needed to compensate the net negative charge in the *x* ∈ ⟨*l*_1_,*l*_2_] region of EDL. This result is not possible in the classical PB approach for a rigid wall model of the interface, where only a smooth decay of counterions’ concentration is possible. This non-classical behavior that our model predicts is the direct consequence of the structural and entropical upgrade of the interface. For comparison, in Figure S10 we have plotted results of Gouy–Chapman model calculations together with the rigid rotor model presented in this work (see Supporting Information). The general difference is the fact that the head groups’ charged segments contribute to the screening of the surface. In Figure S10, we can see that the middle positive charge of the head group lowers the electrostatic potential at the surface, while screening at longer distance is reduced since the terminating negative-charged segment protrudes in the dielectric medium. In the Gouy–Chapman model, electrostatic potential decays to 0 at closer distances.

On the contrary, for the comparison, the study of weakly charged surface *a* = 6 nm^2^ (dashed lines), which corresponds to σ ≈ 0.03 C m^–2^, the decay of Ψ and counterion profiles is smooth, and the conditional probability density is a monotonic increasing function of *x*. The reduced electric field strength at the interface causes less efficient screening by the middle charge of the rigid rotor (i.e., reduced Coulomb attraction). As a result, rigid rotors are preferentially orientated toward the bulk liquid, where the repulsion between the interface and the terminating negative charges is minimal. The theoretical predictions are in good agreement with Monte Carlo simulations for all cases. The case of low surface charge density (high *a*) shows that, in fact, a simple PB approach can be used, instead of more complex models like we presented. Nevertheless, these values of surface charge density or area per lipid molecule are seldom encountered in real systems, if ever.

**Figure 4:**
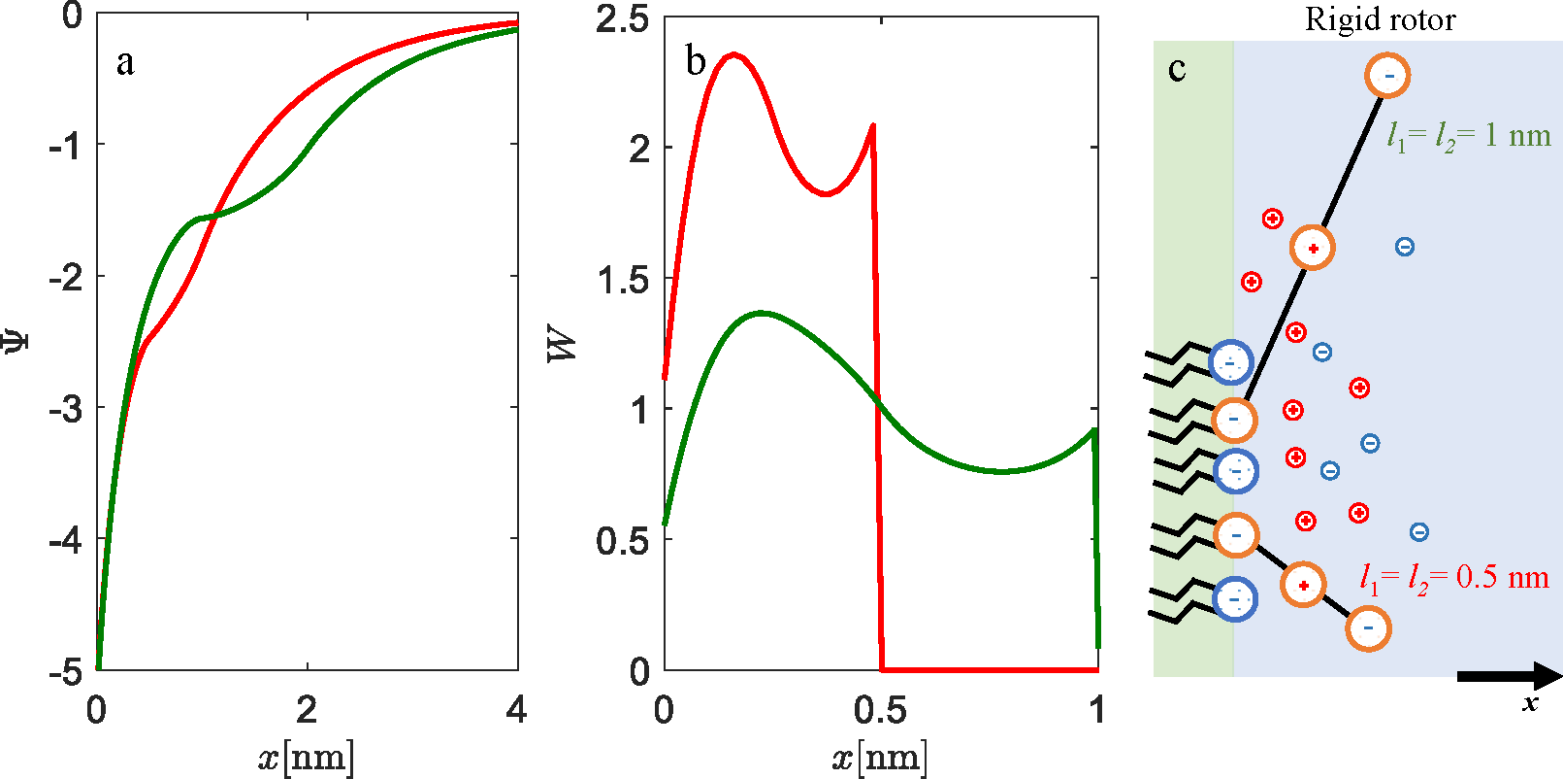
Influence of the length of head groups on EDL properties: (a) electrostatic potential profiles Ψ, (b) probability densities of rigid rod-like head groups *W*, and (c) the schematic representation of the most probable conformation of the rigid rod-like head groups. The lengths of head group segments are *l*_1_ = *l*_2_ = 0.5 nm (red lines) and *l*_1_ = *l*_2_ = 1 nm (green lines). Calculations are made for *c* = 0.1 mol dm^–3^ 1:1 electrolyte concentration, *a* = 0.6 nm^2^, and β = 0.5.

The influence of the length of the head groups on the EDL properties in an aqueous solution of 1:1 salt is presented in Figure 4. Calculations were performed for *a* = 0.6 nm^2^ and concentration of salt *c* = 0.1 mol dm^–3^. Results point to the fact that, in the case of *l*_1_ = *l*_2_ = 1 nm (green lines), the screening of the interface by the middle charge is higher (see Figure 4a) compared to the case of *l*_1_ = *l*_2_ = 0.5 nm (red lines), since Ψ is higher (less negative) near the interface. The corresponding probability density *W*, presented in Figure 4b, shows that *l*_1_ = *l*_2_ = 1 nm is tilted toward the interface, as the maximum is already at around *x* = 0.22 nm (which is 22% of the full extension of the middle charge). In contrast, the case of shorter lengths, *l*_1_ = *l*_2_ = 0.5 nm (red lines), has its maximum in *W* at *x* = 0.17 nm (which is 34% of the full extension of the head groups). The origin of this result lies in the influence of the terminal charge of the head groups. For *l*_1_ = *l*_2_ = 0.5 nm, the terminal charge experiences electric field exhibited by the the interface and is therefore depleted from it by repulsive Coulombic interactions. For *l*_1_ = *l*_2_ = 1 nm, the terminal charge is at larger distances (see Figure 4c).

Beside the surface charge density given by the average area per lipid at the interface *a*, EDL properties, especially in the interfacial region, are strongly dependent on the mole fraction β of the non-structured lipid head group in the monolayer. In Figure S3 in the Supporting Information, conditional probabilities were calculated and simulated for the system variables as in Figure 4 for *l*_1_ = *l*_2_ = 0.5 nm at different β values. On increasing β, the most probable orientation of the middle charge changes from mostly close to the interface toward the perpendicular. The mechanism for this is strong depletion by repulsion between the terminal charge and the interface.

In the special case when the surface charge density is low (*a* = 6 nm^2^), increasing salt concentration causes rigid rotors to tilt toward the interface due to the efficient screening (see Figure S4 in the Supporting Information). Note that this effect cannot be seen for the high surface charge regime.

At this point it is necessary to emphasize that this result is a consequence of the approximation that ions and head group constituents are point-like charges within the theory. While MC simulations are in fairly good agreement for this set of model parameters, we do not consider that the finite size of ions or the polarization effect of water causes this strong condensing of the middle charge toward the interface. If the finite size of ions was included in the free energy functional (*F*_ion_ term),^33,40^ lower local counterions densities at the interface would be obtained. As a result, lower screening of the bare surface charge would cause an even stronger electric field on ions in the solution and charged segments of head groups. We envision that Coulomb attractions between the surface and the middle charged segment would increase, but it is difficult to predict the preferential orientation of head groups due to the excluded volume of ions at the interface. On the other hand, to represent local dielectric properties of the aqueous phase, water molecules can be explicitly included as neutral hard spheres,^40^ or as Langevin dipoles^46^ via an additional entropic contribution into the free energy of the system. By averaging over concentration and orientation of Langevin dipoles, the macroscopic dielectric constant of the system could be derived. The inclusion of explicit solvent would, apart from volume exclusion, partially screen the rigid wall charge and lower the attractions with the middle segment, thus probably favoring a more perpendicular orientation of head groups.

The strength of cDFT is in the fact that the inclusion of these effects is straightforward and equilibrium properties can be studied in a wide range of physical conditions.^48^

In the special case of a rigid rotor where the middle segment is neutral, *q*_1_ = 0, the system reduces to the simpler case of zwitterionic lipids. The results of such calculations are presented Figure S2 in the Supporting Information. Different mole fractions of zwitterionic lipids are studied, and the prediction by the theory is in very good agreement with MC simulations.

#### 3.1.1 Characteristic Lengths of Lipid Monolayer Systems

At this point it is important to discuss the characteristic lengths of the system. While the magnitude of the Bjerrum length *l*_B_ is strictly defined, the Debye length *l*_D_ requires a bit clarification for this system. *l*_D_ scales with the square root of bulk concentration of mobile ions, . In the case of a lipid monolayer, we have charged lipid head group segments immersed in that same aqueous solution of mobile ions, but segments themselves have reduced translational entropy due to the fixed connectivity with the interface. What we practically encounter is the following: the lipid head group segments are contributing to EDL with the charges, but there is a translational anisotropy. The local density approximation for this systems has intrinsic constraint due to the connectivities of lipid head groups. Even though we included head group charges into derivation of the system’s charge density via Gauss’s law, the reduced mobility somewhat complicates the calculation of *l*_D_. At 0.1 mol dm^–3^ concentration of salt in the system, *l*_D_ = 1 nm, which is similar to the average extension of a rigid rotor of flexible head groups. Since the most screening is achieved in this interfacial region of head groups, to simplify the discussion when describing properties of EDL, we will use *l*_D_ only for qualitative purpose, but we focus on the length of the lipid head groups as the characteristic length. Indeed, the description of this interfacial region is the most difficult part. As will be demonstrated throughout the text, the size of the head groups (apart from the charge) strongly affects the properties of the formed EDL at the vicinity of the surface, and in most cases the structural properties dictate screening properties.

In the present study, we considered a model of a flat lipid monolayer, although a membrane in an aqueous phase is constantly fluctuating due to thermal noise.^57^ This membrane fluctuation may affect the electrostatic interactions of the membrane. Guttman and Andelman theoretically considered the effect of electrostatic interactions on membrane fluctuations in two-component membranes.^58^

### 3.2 Flexible Lipid Head Groups

For the case of flexible head groups, the first negative charge, *q*_0_ = −1, is fixed at *x* = 0. The middle charge, *q*_1_ = +1, within the head group is fixed at the distance *l*_1_ from the *q*_0_, whereas the terminal charge, *q*_2_ = −1, is fixed at the distance l_2_ from *q*_1_. Unlike the rigid rod-like rotor, the head group can bend at *q*_1_. Calculations are made with distances between charges *l*_1_ = 0.5 nm and *l*_2_ = 0.5 nm. *W*(*x*, *s*) is the probability density function of the head group, where *x* represents the location of the charge *q*_1_ projected onto the *x*-axis and *s* represents the location of the charge *q*_2_ projected on the same axis (see Figure S1 in the Supporting Information). If we integrate the probability density of flexible lipid head groups, *W*(*x*, *s*), over *s*, we get *W*_*x*_. *W*_*s*_ is obtained by integrating *W*(*x*, *s*) over *x*.

**Figure 5:**
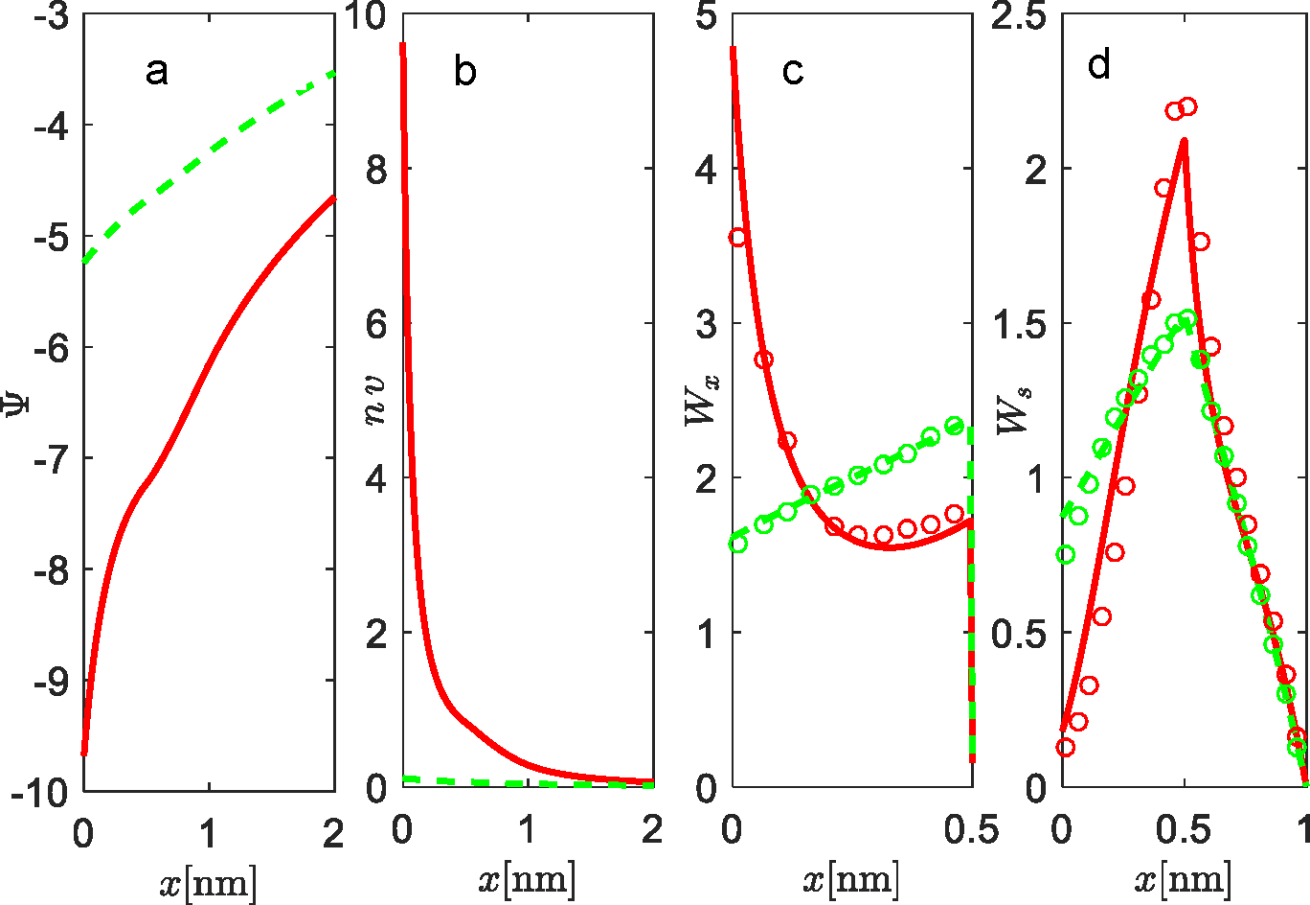
Properties of EDL and the structure of lipid monolayer with flexible head groups in contact with counterions only: (a) electrostatic potential profiles Ψ, (b) concentration profiles of counterions *n*, (c) the probability density, which refers to the position of the middle charge in the head group *W*_*x*_, and (d) the probability, which refers to the position of the terminated charge of the head group *W*_*s*_. The results are shown for two different surface areas per lipid molecules: *a* = 0.6 nm^2^ (full red lines) and *a* = 6 nm^2^ (dashed green lines). MC simulation results are shown by empty circles, red for *a* = 0.6 nm^2^ and green for *a* = 6 nm^2^. The system is electroneutral.

The flexibility of the head groups has a large influence on the structure of the EDL. Namely, the head group flexibility with terminal charge contributes to an additional entropic contribution in the free energy. Figure 5 shows the properties of EDL for the lipid monolayer in contact with a solution of counterions only. The results of theory and MC simulation are presented for two different surface areas per lipid molecule, *a* = 0.6 nm^2^ (full red lines) and *a* = 6 nm^2^ (dashed green lines), and are in excellent agreement with the theory.

For the high-charge regime of the interface (average value of lipid surface per molecule *a* = 0.6 nm^2^), both the theory and MC simulations predict a steep increase of Ψ with *x* in Figure 5a. *W*_*x*_ shows the abrupt probability density increase of the middle charge with the approach to the interface (low *x*). Calculated probability density *W*_*s*_ shows that the terminal charge is also slightly oriented toward the interface, but fully perpendicular orientation is possible as well (see Figure 5d). As a result, a dense layer of counterions that more effectively screen the interface is formed.

It is worth stressing again that, for low charge densities of the interface (*a* = 6 nm^2^), differences between the flexible structure and the rigid rotor are not so evident for the middle charge, and Ψ behaves in classical PB manner. Again, we note that, for those conditions, a simple PB approach without the upgrades of the interface is adequate.

**Figure 6:**
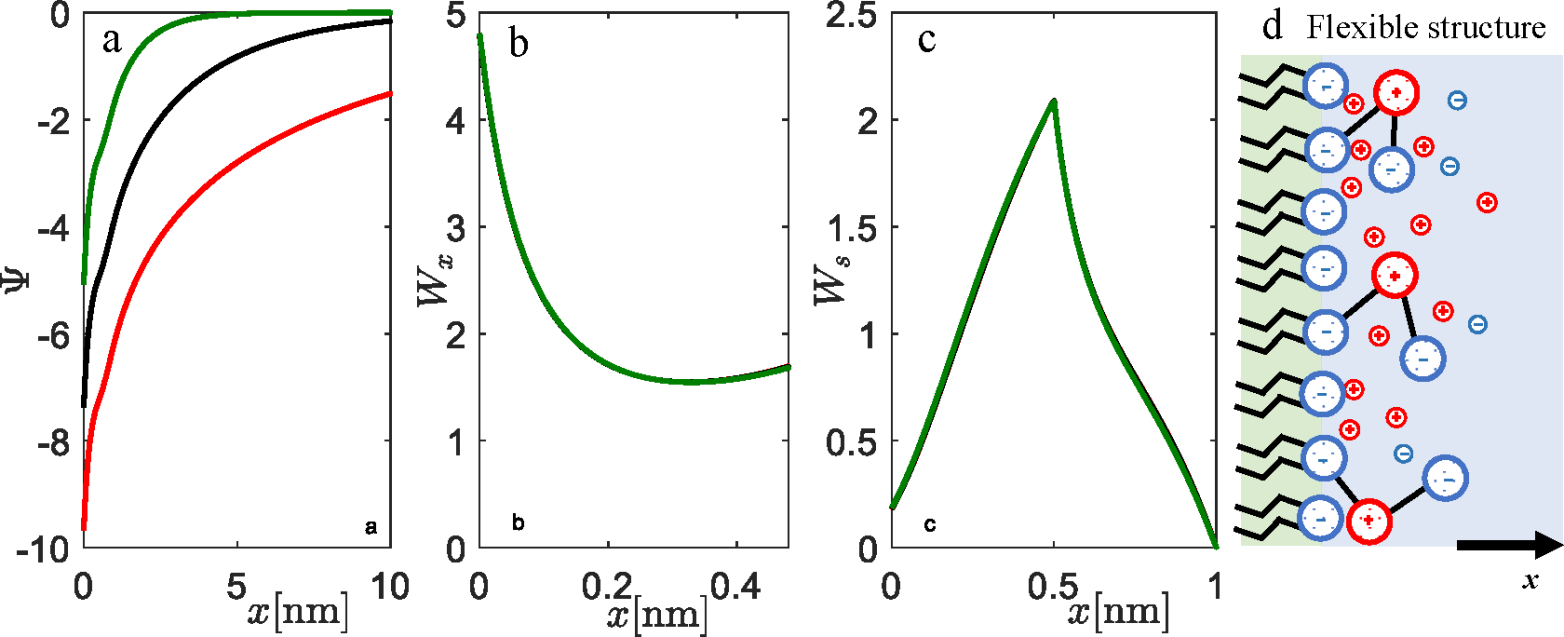
Influence of the salt on EDL and the structure of lipid monolayer with flexible lipid head groups: (a) electrostatic potential, (b) probability, which refers to the position of the middle charge *W*_*x*_, (c) the probability which refers to the position of the terminated charge of the head group *W*_*s*_, and (d) most probable conformations of flexible head groups. The results are shown for three 1:1 salt concentrations, *c* = 0.001 mol dm^–3^ (red line), *c* = 0.01 mol dm^–3^ (black line), and *c* = 0.1 mol dm^–3^ (green line). The surface area per lipid molecule is set to *a* = 0.6 nm^2^, and β = 0.5. The model parameters are *q*_0_ = −1, *q*_1_ = 1, *q*_2_ = −1, *l*_1_ = 0.5 nm, *l*_2_ = 0.5 nm, and *l*_B_ = 0.7 nm.

In Figure 6, we show calculations for different salt concentrations in the system in the relatively high charge regime at *a* = 0.6 nm^2^. As can be seen in Figure 6b,c, the salt does not play an important role in the conformation of flexible lipid head groups. Naturally, it affects the properties of the EDL in terms of its efficiency to screen the interface. The higher screening efficiency with increasing salt concentration is a general consequence of the PB equation. As for the case of counterions only, the positive middle charge is attracted toward the interface, while the negative terminal charge is more perpendicular and localized such that repulsion is minimized.

It is natural to show *W* as a function of both *x* and *s* in three-dimensional plots. Results are presented for counterions only and with salt in Figures S5 and S6, respectively, in the Supporting Information. The domain of *x* covers only the interval between 0 and *l*_1_, whereas the domain for *s* is the interval (0, *l*_1_ + *l*_2_). The peak in the distribution is reached close to *x* = 0, which means that the middle charge is located close to the charged surfaces. The second charge is stretched more to the interior of the liquid, and *W* reaches the maximum at *s* = 0.5 nm. This result is the same for both counterions and salt systems. Since we are in a high charge regime, again the orientation of flexible head groups is unrelated to salt concentration.

Further analysis of EDL properties at all salt concentrations shows that the interfacial region is shorter than the full extension of the flexible lipid head group (*l*_1_ + *l*_2_). The difference between the full extension of the head group and the equilibrium state predicted by the model is around 0.3 nm. This value is comparable to bare ion sizes and the Stern layer, and thus highly relevant in the context of ion association in lipid bilayers.

The case of the low charge of the interface (*a* = 6 nm^2^) with the salt is presented in Figure S7 in the Supporting Information. The large amount of salt strongly screens the negatively charged surface, and the surface becomes effectively more positively charged. Consequently, the conditional probability density shows that the middle part of the head groups is sightly oriented more perpendicular to the charged surfaces. With the decreasing salt concentration, the tendency of head group orientation is more parallel to the charged surface. The main reason is the electrostatic attraction between the positively charged middle groups and the negatively charged surfaces. In the limiting case of very low salt concentration, the results converge to the case of counterions only.

A comparison between the rigid rotor and the flexible structure (both models apply to lipids such as PS) is presented in Figure S8 in the Supporting Information. For medium to high salt concentrations (*c* = 0.01 mol dm^–3^ and higher), high surface charge density (*a* = 0.6 nm^2^), and short segment lengths (*l*_1_ = *l*_2_ = 0.5 nm), the differences in calculated electrostatic potentials and ion distributions are small but not negligible. At *x* = 0.5, the difference in free energy is around 0.3 *k*_B_*T* (see Figure S8a,b), which does not make a drastic change in the association constant of the ions. Nevertheless, for smaller charges of interfaces, as well as for larger segment lengths, the differences between the rigid rotor and flexible structure become more important and originate from the fact that the middle charge of the flexible head group approaches the interface and participates in the screening since the terminal charge is pointed more perpendicular toward the bulk aqueous solution. The rigidness of the rigid rotor does not allow for such participation in screening, since that would require that the terminal negative charge approaches the highly negatively charged interface (see Figure S8c).

Once again, we stress that these results are due to the fact that the current version of the model treats ions as point-like charges; thus, it is necessary to see the difference in the average thickness of the interfacial region when the size of ions and solvent polarization are included within calculations. Also, if the model is to be expanded to bilayer structures, the effect of image charges needs to be included.^59,60^

### 3.3 Triangular Lipid Head Groups

In the case of triangular lipid head groups (PIP_2_), the fixed angle between two negative terminating charges is α = 30°, the length of the head groups is *l* = 0.68 nm, and β = 0.5. The charge numbers are *q*_0_ = −1, *q*_1_ = −1, and *q*_2_ = −1. The probability density, *W*(*x*), corresponds to the projection of the center of mass of both charged moieties to be located at *x* (see Figure S1 in the Supporting Information).

**Figure 7:**
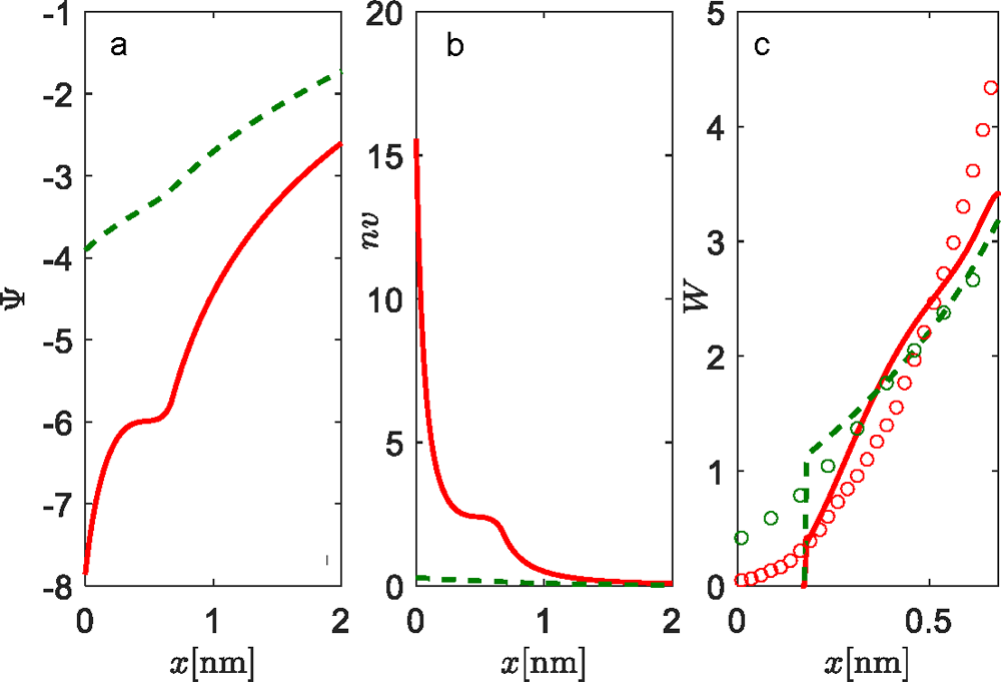
Properties of EDL and the structure of the lipid monolayer with triangular head groups in contact with counterions only: (a) electrostatic potential profiles Ψ, (b) concentration profiles of counterions *n*, and (c) probability densities of triangular head groups *W*. The length of the head groups is *l* = 0.68 nm. The fixed angle between two negative terminating charges is α = 30°, and β = 0.5. The charge numbers are *q*_0_ = −1, *q*_1_ = −1, and *q*_2_ = −1. The system is electroneutral. Results of MC simulations are presented as empty circles. Results are given for two surface areas per lipid molecule: *a* = 0.6 nm^2^ (full red lines and red circles) and *a* = 6 nm^2^ (dashed green lines and green circles).

To study the properties of the EDL in contact with a solution of counterions only, again we examine two distinct cases (see Figure 7): the high charge regime for the surface area per lipid molecules equal to *a* = 0.6 nm^2^ (full red lines), and the low charge regime for *a* = 6 nm^2^ (dashed green lines).

For the case of *a* = 0.6 nm^2^, the electric field is strong and the screening of the interface by counterions is efficient, as can be seen by the steep increase of electrostatic potential at low *x* (Figure 7a,b). This regime in screening is a property of the interfacial region up to the full extension (*l* cos(α/2) ≈ 0.65 nm) of the triangle head groups, which are oriented perpendicular to the interface, toward the bulk solution (see Figure S9 in the Supporting Information). The non-classical “saddle” in screening that occurs just before *l* = 0.68 nm depicts the position of the center of mass of two charges, *q*_1_ = −1 and *q*_2_ = −1. At distances larger than the “saddle” region of *x*, the classical PB screening takes hold. The low charge regime, *a* = 6 nm^2^, shows, as usual, classical PB screening.

The conditional probability density graph (Figure 7c) shows zero probability for angles where the projection of the center of mass falls to *x* = *l* sin(α/2) ≈ 0.175 nm, due to the restriction that the head group cannot penetrate the impermeable lipid rigid wall (the approximation of lipid monolayer at *x* = 0 nm). On the other hand, MC simulations show a non-zero probability density (red and green circles in Figure 7c), even at the interface. This is a consequence of the fact that, in MC simulations, triangle head groups can rotate in 3D (see snapshot of simulations in Figure S9 in the Supporting Information), whereas the theory is valid in 2D. Nevertheless, in both the theory and MC simulations, the probability density increases monotonically with increasing *x*, which suggests that, for the high charge regime, the most probable orientation is perpendicular to the interface (points toward the bulk solution).

**Figure 8:**
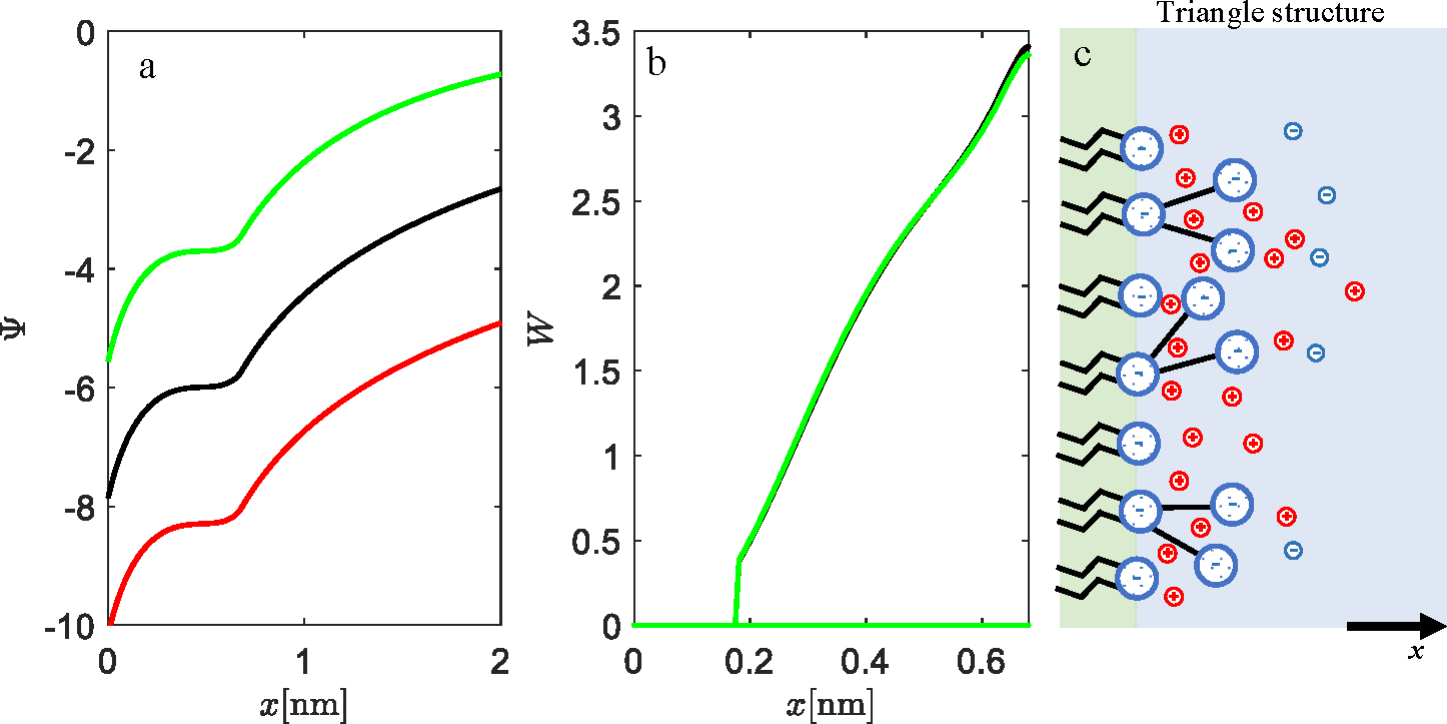
Influence of the salt on EDL and the structure of the lipid monolayer with triangular head groups: (a) electrostatic potential, (b) probability, which refers to the projection of the center of mass of both charge moieties, *W*, and (c) most probable conformations of triangular head groups. The results are shown for three 1:1 salt concentrations, *c* = 0.001 mol dm^–3^ (red line), *c* = 0.01 mol dm^–3^ (black line), and *c* = 0.1 mol dm^–3^ (green line). The surface area per lipid molecule is set to *a* = 0.6 nm^2^, the fixed angle between two negative terminating charges is α = 30°, the length of the head groups is *l* = 0.68 nm, and β = 0.5. The charges are *q*_0_ = −1, *q*_1_ = −1, and *q*_2_ = −1.

If we add salt to the system of high surface charge *a* = 0.6 nm^2^, instead of counterions only (see Figure 8), a similar trend in screening of the surface charge is obtained. Still, due to the co-ions, the effect is less radical, as can be seen in the less steep increase in the electrostatic potential. The probability density shows again that the preferential orientation is perpendicular to the bulk solution (see Figure 8b,c). The salt concentration does not have an important effect on the conformation of lipid head groups, as can be seen from the complete overlapping of curves in Figure 8b. This is a consequence of the strong repulsion between surface charges *q*_0_ = −1 and two terminal head group charges, *q*_1_ = −1 and *q*_2_ = −1. Counterions cannot really weaken this strong repulsion. For this case of a condensed interfacial layer (up to *x* ≈ 0.65 nm), we believe that this effect might hold even if the theory is upgraded for the finite size of ions or water polarization effects. If close contact prevents accumulation of counterions or solvent molecules near the interface, the strong Coulomb repulsion is present, which sets the triangle lipid head groups in the orientation perpendicular to the charged monolayer.

The fact that the triangle groups of PIP_2_ are predominantly perpendicular to the interface generates a strong electric field, which can explain the condensation of positively charged macro-ions to PIP_2_- or PIP_3_-rich domains.^23,61^

## 4 Conclusion and Outlook

In the present work we proposed a model of a multicomponent lipid monolayer in contact with an aqueous solution of ions. The description of the interface properties is upgraded within the cDFT framework. The interface is made of structured charged head groups with conformational and orientational degrees of freedom. We focused especially on rigid rotors, flexible structures, and triangular structures. It must be emphasized that the model does not consider partitioning of ions in the interfacial region as a fitted quantity or as a Stern layer. Instead, it has a discrete description of head groups, and ions can freely penetrate between the interface (close to a rigid wall), middle, and terminal charged segments. Screening of the charge, different from the usual Poisson–Boltzmann approach, is thus achieved without parametrization.

The theory was systematically compared to MC simulations. For a wide range of system conditions, a very good agreement was obtained, even in the case when non-classical EDL properties are predicted. To demonstrate the generality of the method, within this paper we considered only a 1:1 mol ratio mixture of lipids with a complicated charge structure versus the simple charged lipids that only contribute to the charge of the interface. Nevertheless, the framework is general, and different lipid mole fractions can be studied easily. Furthermore, the variety of common types of lipid monolayers can be represented by changing the charge numbers and the structure of lipid head groups within the free energy functional (see details in the Model and Methods section).

The results show that the lipid head group structure substantially disrupts the EDL in the vicinity of phosphate monolayer. For the head groups that have a middle charge opposite to the charge of the interface, the electrostatic attraction causes a more parallel orientation with respect to the interface. When comparing a rigid rotor and the flexible structure, the conditional probability density shows that the flexibility of head groups allows even further approach of the middle charge toward the interface, while the terminal charge is more stretched perpendicular toward the bulk solution to minimize the repulsion with the negative interface.

Triangular head group are preferentially oriented perpendicular to the interface due to the strong repulsion between the two negative terminal charges and the negative interface, and the effect persists throughout the range of surface areas or salt concentrations.

In future works we are going to study the thermodynamics of different head groups on the phase separation between different type of lipid head groups. We assume that the phase separation is basically suppressed in lipid membranes containing unsaturated, negatively charged lipids compared to neutral (zwitterionic) lipid membranes. In addition, the membrane morphology is also affected by the presence of charged lipid.^62^ From these experiments, it has been reported that the electric charges on the hydrophilic head groups have large effects on the formation of phase separation and membrane morphology, and some theoretical and numerical simulation studies have been successful in giving qualitative explanations to the experimental results.^63−66^ In this sense, our group is currently using our newly developed theory to study the effects of in-plane phase separation for varying mole ratios of charged and zwitterionic lipids within the Bragg–Williams approximation. Additionally, the finite size of ions and water polarization effect will be introduced to further rationalize the effect of overlapping lipid membranes’ EDLs. Also the first attempt at association/adsorption of multiple ions onto sites which are not in-plane but pointing into the aqueous solution will be included within the cDFT framework as modified charge regulation where different association sites are modeled explicitly.^56^

## Supporting Information Available

Schematic representation of projections of characteristic elements onto *x*-axis for the three head groups types; results for zwitterionic lipid head groups as a simplified case of rigid rotor; orientations of rigid rotor for different mole fractions in the monolayer, and for low interface charge; 3D graphs of the probability density for projections of both middle and the terminal charges of flexible structures; comparison of the rigid rotor and the flexible structures for negatively charged lipids; MC simulations snapshots for triangular lipid headgroup (PDF)
