# Supplementary material for: Influence of Charge Lipid Head Group Structures on Electric Double Layer Properties
Source: J Chem Theory Comput. 2021 Dec 22;18(1):448–60. doi: 10.1021/acs.jctc.1c00800 (PMC8757465; doi:10.1021/acs.jctc.1c00800)
Supplement: Supplementary file 1 — ct1c00800_si_001.pdf [file ct1c00800_si_001.pdf]

Supporting Information for: Influence of Charge Lipid Head Group Structures on Electric Double Layer Properties

Klemen Bohinc,*,† Mario Špadina,† Jurij Reščič,‡ Naofumi Shimokawa,¶ and Simone Spada*,§

†*Faculty of Health Sciences,University of Ljubljana, SI-1000 Ljubljana, Slovenia*

‡*Faculty of Chemistry and Chemical Technology, University of Ljubljana, Večna pot 113, SI-1000 Ljubljana, Slovenia*

¶*Japan Advanced Institute of Science and Technology, 1-1 Asahidai, Nomi, Ishikawa 923-1292 Japan*

§*National Institute of Oceanography and Applied Geophysics - OGS, Italy*

E-mail: klemen.bohinc@zf.uni-lj.si; simonespada.info@gmail.com

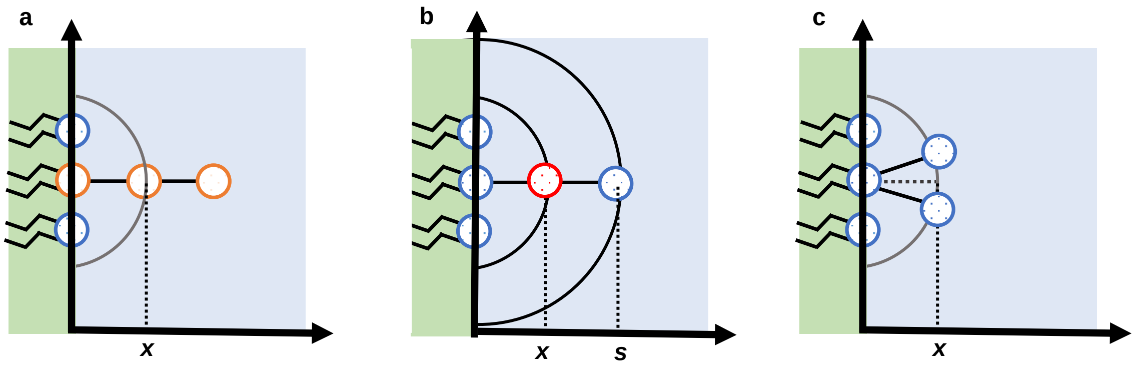

Figure S1: Projections of designated charges of the three lipid head gorups. a) In the case of the rigid rotor, the projection on x-axis refers to the middle charge, b) in the case of flexible structure, the additional degrees of freedom require two projections on x-axis, namely of middle x and terminating s charges, and c) in the case of triangle structure the projection of the center of the mass of two charges is computed on the x-axis.

Rigid rotors

Fig. S3a shows theoretical conditional probability densities of head groups W as a function of x for different fractions of charged lipids β. The increasing fraction of rigid rotors causes less non-homogeneous distribution of W, suggesting more perpendicular orientation towards the bulk solution. Fig. S3b shows conditional probability densities of head groups W as a function of x obtained by MC simulations. The probability densities obtained by MC simulations qualitatively follow the results of the theory. The quantitative discrepancy is the result of finite size of charges which needs to be accounted for in MC simulation. On contrary in the theory the charges are point-like.

The influence of added salt on the properties of EDL is shown in Fig. S4. In the limiting case of very low salt concentration the results converge to the case of counterions only. In the case of only counterions the conditional probability density shows that the rigid head groups are oriented more perpendicular to the charged surfaces. With increasing salt concentration the tendency of head group orientation is more parallel to the charged surface. The main reason is the increased screening which effectively increases with increasing salt concentration.

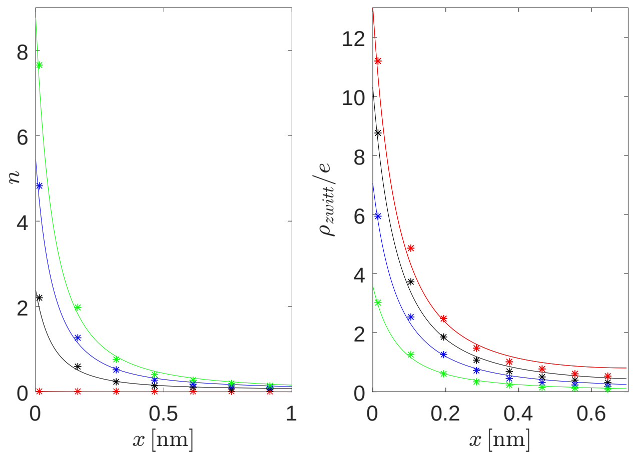

Figure S2: Zwitterionic lipid head groups are composed only of two charges. The phosphate groups are located at $x = 0$, whereas the positively charge moiety of amino group can freely rotate around the phosphate group. The length of the head groups is $l = 0.7$ nm Left: concentration profiles of counterions. Right: charge density profiles of the head groups. The ratio of simple charged head groups (lipids with only phosphate group) and zwitterionic lipids: red - 0%, black- 25%, blue -50% and green -75%.

Flexible structure

In the case of flexible lipid head groups the probability density W depends on two variables x and s. Therefore it is natural to show three dimensional plots. Figs. S5 and Fig. S6 show a three dimensional plots of the probability density W as a function of x and s. The domain of s covers only the interval between 0 and l_1 whereas the domain for s is the interval $(0, l_1 + l_2)$. The peak in the distribution is reached close to $x = 0$ which means that the middle charge is located close to the charged surfaces.

The second charge is stretched more to the interior of the liquid and W reaches the max-

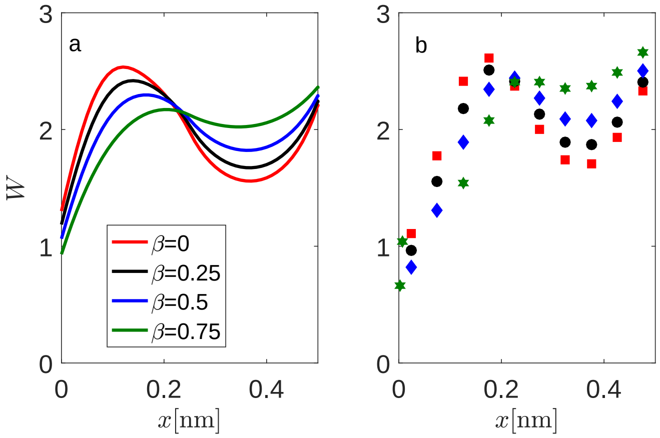

Figure S3: Calculations for four different mole fractions of rigid rotors lipids: $\beta = 0$ (red curve and squares), $\beta = 0.25$ (black curve and circles), $\beta = 0.5$ (blue curve and diamonds) and $\beta = 0.75$ (green curve and stars). Theoretical conditional probability density profiles (a) and MC simulations (b) are shown. The model parameters are $a = 0.6\,\mathrm{nm}^2$, $l_1 = 0.5\,\mathrm{nm}$, $l_2 = 0.5\,\mathrm{nm}$, $l_\mathrm{B} = 0.7\,\mathrm{nm}$, $q_0 = -1$, $q_1 = 1$ and $q_2 = -1$.

imum at $s = 0.5\,\mathrm{nm}$. Fig. S5 corresponds to the electrolyte solution composed of counterions only whereas Fig. S6 corresponds to the electrolyte which includes salt.

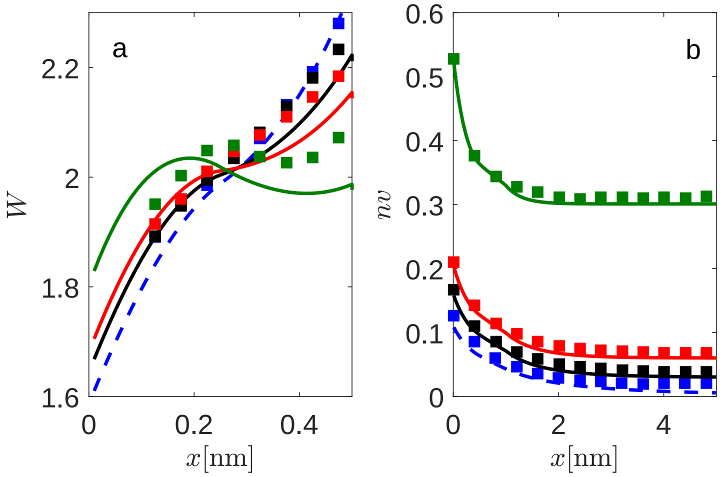

Figure S4: Rigid rotors case at low charge regime of the interface for $a = 6$ nm^2 ($\sigma \approx 0.03$ C m^{-2}) and the influence of added salt. Theory (lines) and MC simulations (squares) are presented for the cases: counterions only (black dashed), 0.05 mol dm^{-3} (black), 0.1 mol dm^{-3} (red) and 0.5 mol dm^{-3} (green) a) Conditional probability density profiles W of head groups, b) counterion concentration profiles n. The model parameters are $a = 6\,\text{nm}^2$, $l_1 = 0.5\,\text{nm}$, $l_2 = 0.5\,\text{nm}$, $\beta = 0.5$, $l_\text{B} = 0.7\,\text{nm}$, $q_0 = -1$, $q_1 = 1$ $q_2 = -1$.

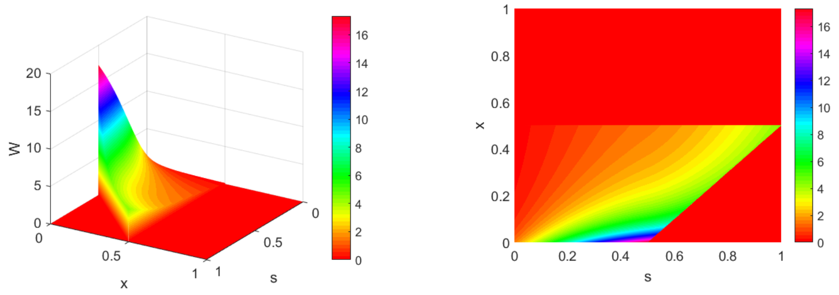

Figure S5: Flexible lipid head groups and couterions only. The conditional probability density W as a function of x and s is shown. The right graph shows the projection of W on the (x, s) plane. The model parameters are $q_0 = -1$, $q_1 = 1$ $q_2 = -1$, $l_1 = 0.5\,\text{nm}$, $l_2 = 0.5\,\text{nm}$, $l_\text{B} = 0.7\,\text{nm}$ and $a = 0.6\,\text{nm}^2$.

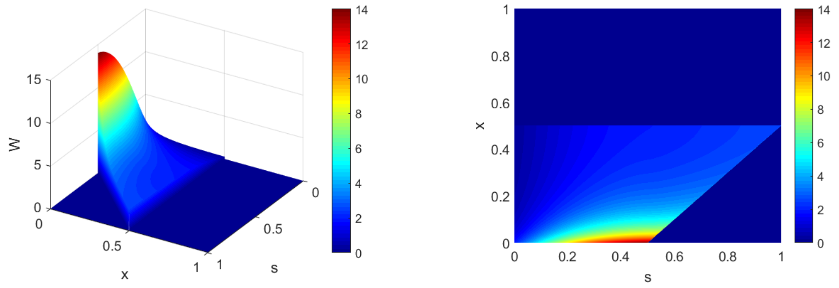

Figure S6: Flexible lipid head groups with salt. The conditional probability density W as a function of x and s is shown. The right graph shows the projection of W on the (x, s) plane. The model parameters are $q_0 = -1$, $q_1 = 1$ $q_2 = -1$, $l_1 = 0.5\,\text{nm}$, $l_2 = 0.5\,\text{nm}$, $l_B = 0.7\,\text{nm}$, 0.1 mol dm^{-3} salt and $a = 0.6\,\text{nm}^2$.

The influence of added salt on the properties of flexible lipid head groups is shown in Fig. S7. The large amount of salt strongly screens the negatively charged surface and the surface becomes effectively more positively charged. Consequently the conditional probability density shows that the middle part of the head groups is sightly oriented more perpendicular to the charged surfaces. With the decreasing salt concentration the tendency of head group

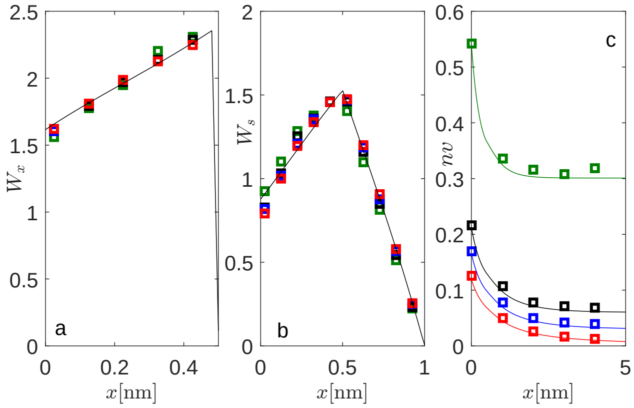

Figure S7: Flexible structure monolayer at low charge regime of the interface for $a = 6$ nm^2 ($\sigma \approx 0.03$ C m^{-2}) and the influence of added salt) Conditional probability density profiles W_x of head groups, b) Conditional probability density profiles W_x of head groups and c) counterion concentration profiles n. Influence of added salt on flexible lipid head groups: 0.005 mol dm^{-3} (blue curves), 0.05 mol dm^{-3} (black curves), 0.1 mol dm^{-3} (red curves), and 0.5 mol dm^{-3} (green curves). MC simulations are presented by squares. In a) and b) the theoretical results are shown by only black lines due to very similar results. Mixture of structured flexible lipids and simple negatively charged lipids is $\beta = 0.5$. The model parameters are $a = 6\,\mathrm{nm}^2$, $l_1 = 0.5\,\mathrm{nm}$, $l_2 = 0.5\,\mathrm{nm}$, $l_B = 0.7\,\mathrm{nm}$, $q_0 = -1$, $q_1 = 1$ $q_2 = -1$.

orientation is more parallel to the charged surface. The main reason is the electrostatic attraction between the positively charged middle groups with the negatively charged surfaces. In the limiting case of very low salt concentration the results converges to the case of counterions only.

Triangular Lipid Head Groups

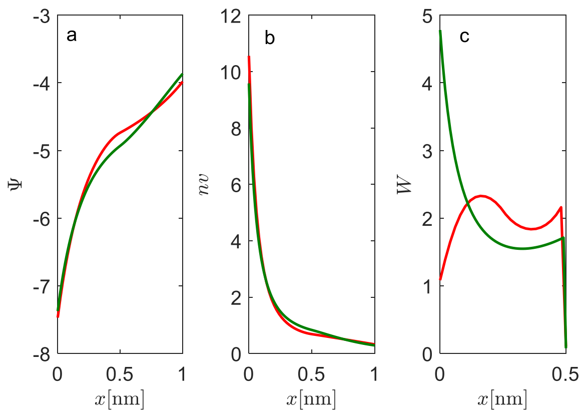

Figure S8: Comparison the properties of EDL for the rigid rotor (red line) and the flexible structure (green line). a) Electrostatic potential, b) counterions profiles, and c) probability which refers to the position of the middle charge W. The results are shown for $c = 0.01$ mol dm^{-3} of 1:1 electrolyte, the surface area per lipid molecules is set to $a = 0.6\,\text{nm}^2$ and $\beta = 0.5$. The model parameters are $q_0 = -1$, $q_1 = 1$, $q_2 = -1$, $l_1 = 0.5\,\text{nm}$, $l_2 = 0.5\,\text{nm}$ and $l_\text{B} = 0.7\,\text{nm}$.

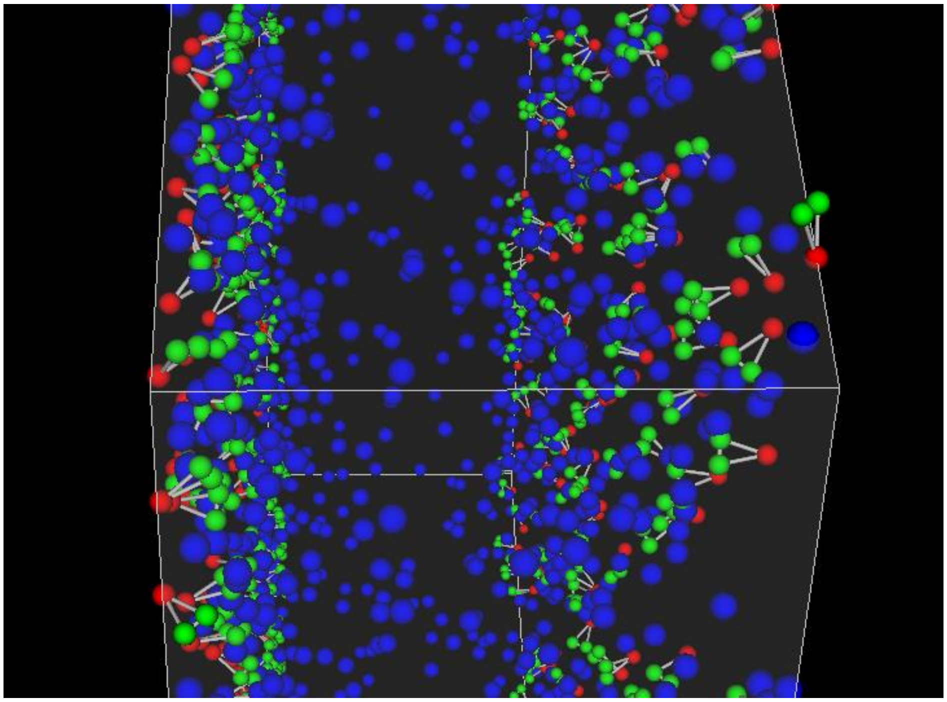

Figure S9: Snapshot of triangular lipid head groups attached to the surface. The red spheres depict the negative phosphate group q_0, while green spheres depict two negative terminating charges. Blue spheres depict mobile counterions. The length of the head groups is l =0.68 nm. the charge numbers are $q_0 = -1$, $q_1 = -1$, $q_2 = -1$. The angle between two terminal charges is $\alpha = 30°$ Results are given for surface area per lipid molecule $a = 0.6$ nm^2. The system is electroneutral.

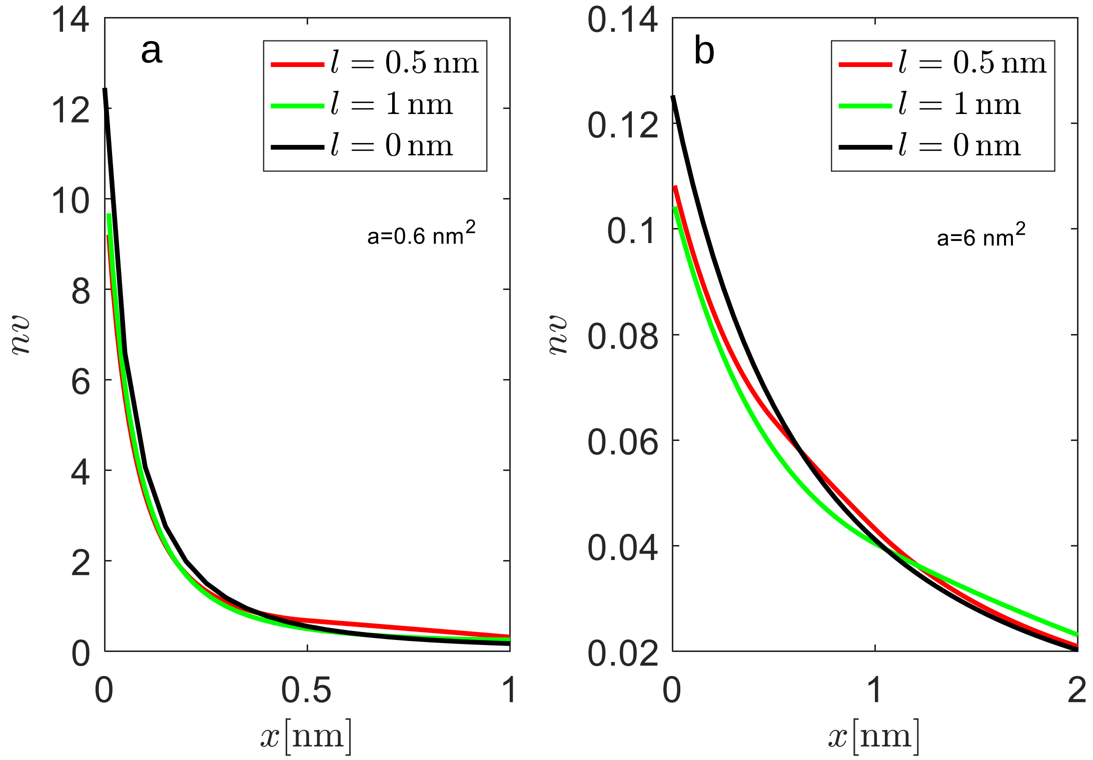

Figure S10: Comparison between the Gouy-Chapman model for flat the interface and the rigid rotor model presented in this work. Concentrations of counterions nv as a function of distance from the interface x. l is a length of rigid rotor head groups segments, $l = 0$ represents the solution of the Gouy-Chapman model. a) surface area per lipid molecule $a = 0.6$ nm^2, and b) surface area per lipid molecule $a = 6$ nm^2. The system is electroneutral.
